# An attenuated rate of leg muscle protein depletion and leg free amino acid efflux over time is seen in ICU long-stayers

**DOI:** 10.1186/s13054-017-1932-6

**Published:** 2018-01-23

**Authors:** Lena Gamrin-Gripenberg, Martin Sundström-Rehal, Daniel Olsson, Jonathan Grip, Jan Wernerman, Olav Rooyackers

**Affiliations:** 1 Division of Perioperative Medicine and Intensive Care, Karolinska University Hospital, Huddinge, Sweden; 2 Anesthesiology and Intensive Care, Department of Clinical Science Intervention and Technology (CLINTEC), Karolinska Institutet, Huddinge, Sweden; 3 Medical Statistics, Department of Learning, Informatics, Management and Ethics, Karolinska Institutet, Stockholm, Sweden

**Keywords:** Skeletal muscle, Protein turnover, 3-Methylhistidine, Multiple organ failure, Stable isotopes, Plasma amino acids

## Abstract

**Background:**

There is extensive documentation on skeletal muscle protein depletion during the initial phase of critical illness. However, for intensive care unit (ICU) long-stayers, objective data are very limited. In this study, we examined skeletal muscle protein and amino acid turnover in patients with a prolonged ICU stay.

**Methods:**

Patients (*n* = 20) were studied serially every 8–12 days between days 10 and 40 of their ICU stay as long as patients stayed in the ICU. Leg muscle protein turnover was assessed by measurements of phenylalanine kinetics, for which we employed a stable isotope-labeled phenylalanine together with two-pool and three-pool models for calculations, and results were expressed per 100 ml of leg volume. In addition, leg muscle amino acid flux was studied.

**Results:**

The negative leg muscle protein net balance seen on days 10–20 of the ICU stay disappeared by days 30–40 (*p* = 0.012). This was attributable mainly to an increase in the de novo protein synthesis rate (*p* = 0.007). It was accompanied by an attenuated efflux of free amino acids from the leg. Leg muscle protein breakdown rates stayed unaltered (*p* = 0.48), as did the efflux of 3-methylhistidine. The arterial plasma concentrations of free amino acids did not change over the course of the study.

**Conclusions:**

In critically ill patients with sustained organ failure and in need of a prolonged ICU stay, the initial high rate of skeletal muscle protein depletion was attenuated over time. The distinction between the acute phase and a more prolonged and more stable phase concerning skeletal muscle protein turnover must be considered in study protocols as well as in clinical practice.

**Trial registration:**

Australian New Zealand Trial Registry, ACTRN12616001012460. Retrospectively registered on 1 August 2016.

**Electronic supplementary material:**

The online version of this article (10.1186/s13054-017-1932-6) contains supplementary material, which is available to authorized users.

## Background

Loss of muscle mass is a well-known phenomenon in critical illness. The accompanying muscle weakness is reported to be related to long-term outcomes [1]. The loss of muscle mass and function is attributed to a combination of general inflammation, anabolic resistance, and immobilization [2]. In parallel, there is critical illness myopathy and/or neuropathy, with clinical diagnostic features, neurophysiologic measures, and histology [3].

Several therapies are suggested and practiced to attenuate the loss of muscle mass and function, embracing the hypothesis that preserving this will smoothen recovery and improve outcomes. Nutritional interventions, on their own or in combination with general mobilization, bed exercise, electric stimulation, and pharmacologic interventions, are all examples of suggested treatment modalities [4].

Measuring changes in muscle mass, as well as characterizing the underlying pathophysiologic mechanisms, is necessary to evaluate these potential treatments. The heterogeneity of diagnoses, comorbidities, and levels of fluid retention add to the complexity of studying muscle loss in critical illness.

During the initial 1–2 weeks of intensive care unit (ICU) stay, there is comprehensive documentation. A loss of muscle mass and muscle proteins of 10–20% is seen, as documented by biochemical analyses of biopsies as well as imaging by ultrasound and computed tomography (CT) [5–8]. The net loss is mediated by an imbalance between de novo synthesis and breakdown of muscle proteins [9–12]. This imbalance seems to be equally distributed among different categories of muscle proteins [13]. In particular, breakdown rates of muscle protein are enhanced [9, 12]. The fractional synthesis rate of muscle is more scattered than in the general population, but it is not different, on average, from normal [14–16]. For the small group of patients in need of prolonged ICU stay, with the risk of an even more profound loss of muscle mass, the documentation is very sparse. We therefore wanted to characterize muscle protein turnover between days 10 and 40 of ICU stay by measuring de novo protein synthesis, protein breakdown, and protein balance of leg muscle together with amino acid balances across the leg, representing skeletal muscle.

## Methods

This study was performed in the general ICU of Karolinska University Hospital in Huddinge, Sweden, during the period of February 2011 to January 2014. Inclusion criteria were sustained organ failure, age ≥ 18 years, ICU stay ≥ 10 days, provision of informed consent, no withholding of treatment, and no contraindication either to inserting a catheter into the femoral vein or to a percutaneous muscle biopsy in the lateral portion the quadriceps femoris muscle. The protocol was approved by the Regional Ethics Committee in Stockholm (2008/642), and informed consent was obtained from the patient or, more commonly, from a next of kin after information on the protocol and the risks involved was provided orally as well as in writing. The protocol was retrospectively registered (Australian New Zealand Trial Registry, ACTRN12616001012460).

The protocol contained a measurement of skeletal muscle protein turnover as outlined below every tenth day (8–12 days) of the ICU stay, as long as the patient was in the ICU and the inclusion criteria were still met, leading to patients being studied serially one (*n* = 10), two (*n* = 9), or three (*n* = 1) times. The different numbers of measurements per patient were due to patients not meeting inclusion criteria on all days, patients who left the unit or died, or logistical reasons because not enough research personnel were available. There was no specific treatment protocol besides the general guidelines of the unit as applied by the acting intensivist. In general, all patients were given continuous feeding, preferably by the enteral route. Also, nutrition was continued during the study. The unit is typical of a general ICU at a university hospital in Scandinavia, with a 1:1 patient/nurse ratio, intensivist present at the unit 24 h/7 days per week, daily assessment of the level of care, active physical mobilization, and daily wake-up test for patients who are sedated. Compared with units in many other parts of the world, there is a relatively low number of ICU beds relative to hospital beds and relative to the population.

The procedure of leg muscle protein turnover measurement has been described in detail before [12]. The protocol began with a baseline sample from the arterial line, after which a primed continuous infusion of [ring-^2^H_5_]phenylalanine (prime 0.5 mg/kg; infusion 0.5 mg/kg/h) and [^2^H_3_]methylhistidine (prime 0.01 mg/kg; infusion 0.01 mg/kg/h) was started and continued for 150 minutes. During this period, patients were at rest, and no alterations in medications or nutrition support were made. Approximately 120 minutes after the baseline sample was collected, a catheter was inserted into the femoral vein with ultrasound guidance. Thereafter blood samples were obtained simultaneously from the arterial line and from the femoral vein at 135, 140, 145, and 150 minutes after baseline. Leg blood flow was measured by venous occlusion plethysmography ≥ 10 minutes before and immediately after the sampling period, with each measurement including ten readings [17]. A muscle biopsy was taken at 150 minutes using a Bergström needle after administration of a local anesthetic to the skin and subcutaneous tissue [6]. Calculations of the blood flow from the plethysmographic readings were performed by an independent person blinded to the study design and time points. Plasma flow was calculated using the individual hematocrit values.

Plasma was obtained from the blood samples by centrifugation and were stored at −80 °C pending analysis. Muscle biopsies were immediately frozen in liquid nitrogen and thereafter stored at −80 °C pending analysis. Muscle samples were freeze-dried and subsequently cleaned from blood and connective tissue using a microscope before analyses. The further procedures for sample handling and analyses of plasma and muscle tissue are described in detail elsewhere [12]. For the calculations of muscle protein kinetics, both a two-pool and a three-pool model were used. Details of these calculations are described elsewhere [12]. The two-pool model uses the net balance of both the total phenylalanine and the labeled phenylalanine over the leg (arterial concentration minus venous concentration times the leg plasma flow) to calculate the rate of appearance and rate of disappearance of phenylalanine from the leg muscle. Because phenylalanine cannot be synthesized or degraded in muscle tissue, these values represent protein breakdown and synthesis, respectively. The main limitation of the two-pool approach is that phenylalanine from protein breakdown recycled within muscle to new protein synthesis is not detected. To overcome this, a measurement of the labeled phenylalanine in a muscle biopsy can be used to correct for this, and in this three-pool model, the real muscle protein breakdown and synthesis rates can be calculated. We used both models to see if both showed the same directional changes and if we would be able to use the less invasive two-pool model in future studies to evaluate interventions.

The calculations of the balances of the individual amino acids, including 3-methylhisitidine, are described in detail elsewhere [17, 18]. 3-Methylhistidine is formed by posttranslational modification of histidine in contractile proteins only. Because humans cannot degrade or reuse 3-methylhistidine, its release is an estimation of the breakdown of contractile protein specifically. For 3-methylhistidine, an isotopic label was used to achieve sufficient sensitivity when using a two-pool model [18]; for all other amino acids, a net balance combined with plasma flow was used. 3-Methylhistidine rate of appearance is a measure of breakdown rates of contractile proteins from skeletal muscle.

The sample size calculation was based on a target of not less than ten patients studied between days 30 and 40 of their ICU stay. On the basis of historic records of the ICU, it was anticipated that inclusion of not less than 30 patients on day 10 would be needed to achieve this target. The statistical plan was to compare values obtained between days 10 and 20 with those obtained between days 30 and 40. For this purpose, the Mann-Whitney *U* test was used (Statistica 10 software; Tibco Software, Chapel Hill, NC, USA). However, one patient was studied in both periods, and only the last measurement was included to make the groups comparable in size. Two patients were studied twice in one period, and for these the first measurement was included in this statistical analysis, resulting in ten observations in the period 10–20 days and nine in the period 30–40 days. In addition, we used a linear mixed effects model to include all measurements (both singular and repeated) and evaluate the changes over time. For each outcome variable, a random intercept model with continuous time as the only explanatory variable was fitted. We used the lme function [19] of the nlme package in R version 3.4.0 software [20]. Residual plots were examined to check if the assumptions were fulfilled. As a sensitivity analysis, patients with a residual larger than ±3 SD on some measurement were removed, and the model was refitted to see if that changed the conclusions in terms of significance at the 5% level. Because no conclusions changed, only the complete data analyses were reported.

## Results

In total, 30 measurements were performed in 20 patients with sustained multiple organ failure on days 10–40 of their ICU stay, with patients studied 1 (*n* = 10), 2 (*n* = 9), or 3 (*n* = 1) times. When the target of ten measurements between days 30 and 40 of the ICU stay was obtained, patient recruitment was stopped. Characteristics of the included patients are summarized in Table 1 and given in more detail in Additional file 1: Tables S1 and S2.

**Table 1:** Patient characteristics

Characteristics	Data
Female sex, *n* (%)	5 (25)
Age, years (IQR)	65 (64–71)
BMI, kg/m^2^ (IQR)	26.0 (23.5–28.9)
Diagnosis	
Medical, *n* (%)	3 (18)
Surgical, *n* (%)	14 (82)
ICU length of stay, days (IQR)	26 (21–32)
SOFA score (IQR)	6 (4–7)
Admission weight, kg (IQR)	82 (73–102)
Caloric intake, kcal/kg/24 h	20 ± 3.2
Amino acid intake, g/kg/24 h	1.0 ± 0.2
Insulin, IU/24 h (IQR)	53 (30–71)

*Abbreviations: BMI* Body mass index, *ICU* Intensive care unit, *SOFA* Sequential Organ Failure Assessment

Values are given as *n* (%), median (IQR), or mean ± SD as appropriate

**Fig. 1:**
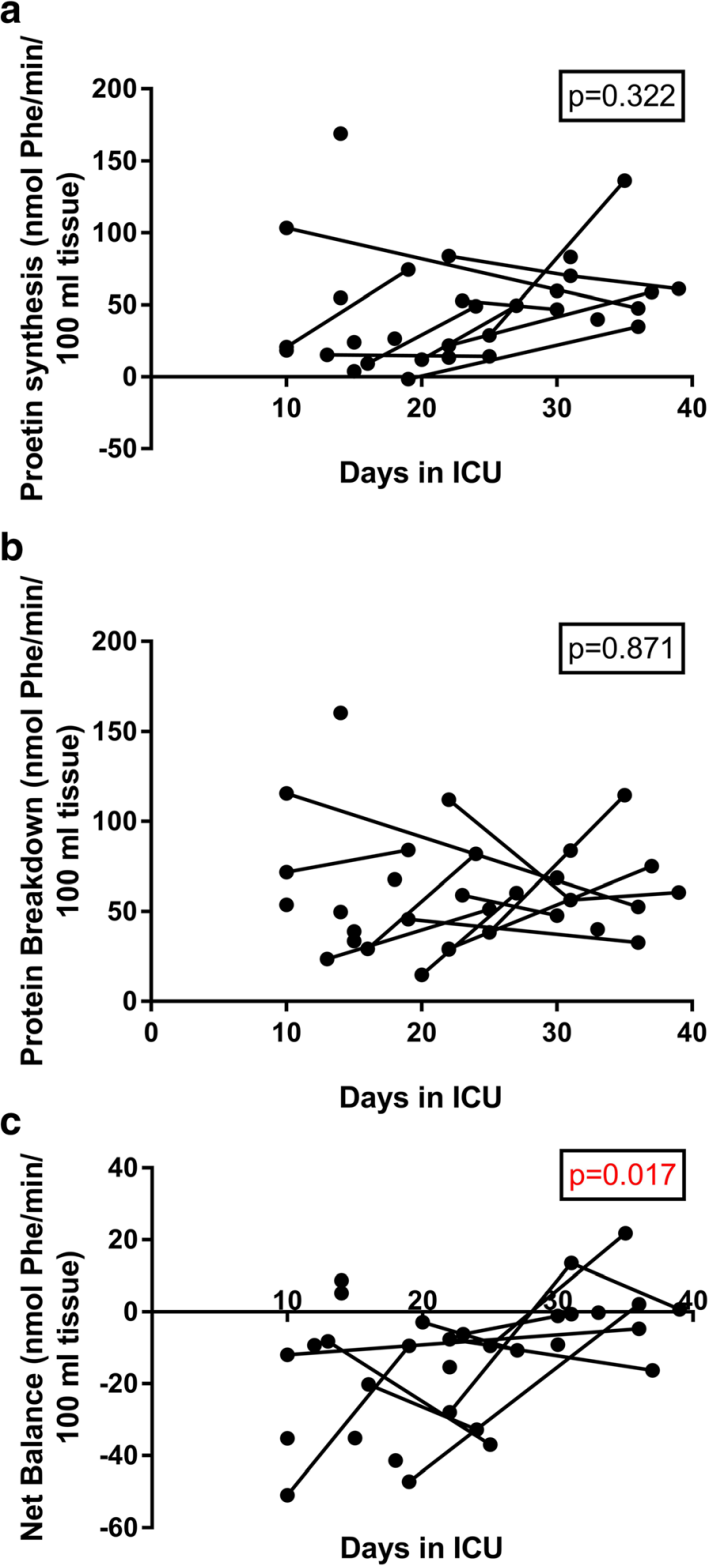
A longitudinal presentation of skeletal muscle mixed protein turnover on 30 occasions in long-staying intensive care unit (ICU) patients (*n* = 20). **a** Protein synthesis. **b** Protein breakdown. **c** Protein net balance. The *p* values from the linear mixed effects model against time in the ICU are indicated

**Fig. 2:**
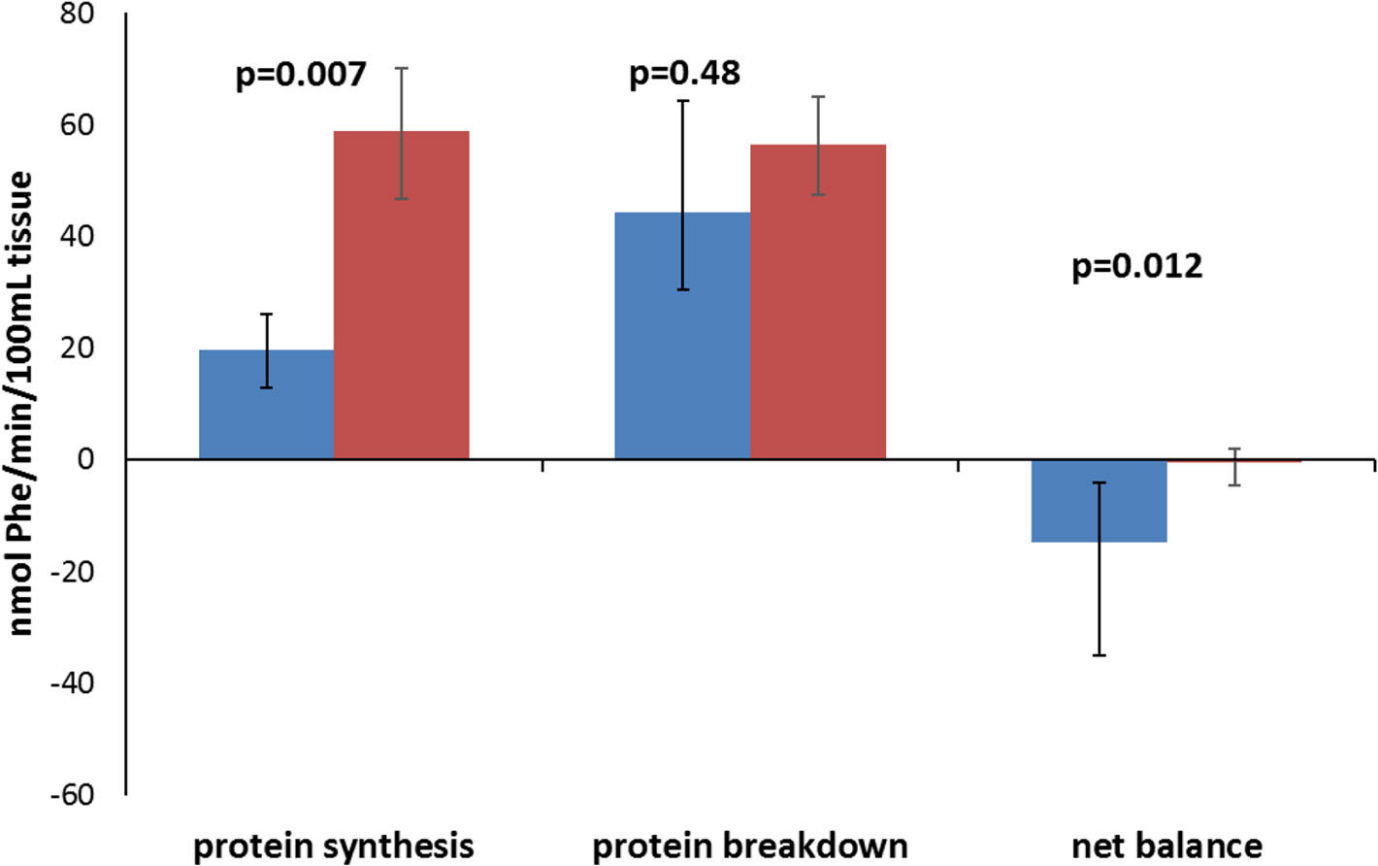
Skeletal muscle mixed protein turnover in long-staying intensive care unit patients (*n* = 20). Measurements in the period days 10–20 (*blue bars*; *n* = 10) are compared with those in the period days 30–40 (*red bars*; *n* = 9). Data are given as medians (quartiles). The *p* values are given for nonparametric comparisons between the two time periods

**Fig. 3:**
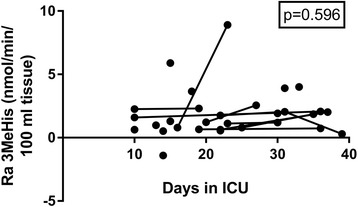
A longitudinal presentation of skeletal muscle contractile protein degradation as reflected in the efflux of 3-methylhistidine from the leg on 30 occasions in long-staying intensive care unit (ICU) patients (*n* = 20). The *p* values from the linear mixed effects model against time in the ICU are indicated

The individual muscle protein turnover data, given as protein synthesis rate, protein breakdown rate, and protein net balance, are depicted in the three panels of Fig. 1. The *p* values for the changes over time are presented in the figure. The results show that muscle protein balance became less negative over time and reached, on average, a zero balance around day 35. This change seems to have been more driven by an increase of muscle protein synthesis over time, although no significant trend was observed, whereas muscle protein breakdown did not change. The calculated plasma flows, given in Additional file 1: Figure S1, did not contribute to the altered net muscle protein balance, because they showed no change over time. Results of the two-pool model are given as rates of disappearance and appearance of phenylalanine in Additional file 1: Figure S2. The changes in the rates of disappearance and appearance showed the same trends as changes in muscle protein synthesis and breakdown from the three-pool model. This implies that future studies investigating clinical or nutritional interventions can use the less invasive two-pool model to study the effects on muscle protein metabolism, thereby enabling more patients to be included in these studies. Figure 2 illustrates the change in the muscle protein turnover variables on days 30–40 of the ICU stay as compared with days 10–20. Specific patient characteristics for these two periods are given in Additional file 1: Table S2. The data showed a more positive muscle protein net balance in the later period of the ICU stay. This alternation in balance was attributable mainly to the higher muscle protein synthesis without any contribution from muscle protein breakdown. Also, the breakdown rates of the myofibrillar proteins, as estimated by the rate of appearance of 3-methylhistidine, did not show a change over time (Fig. 3).

**Fig. 4:**
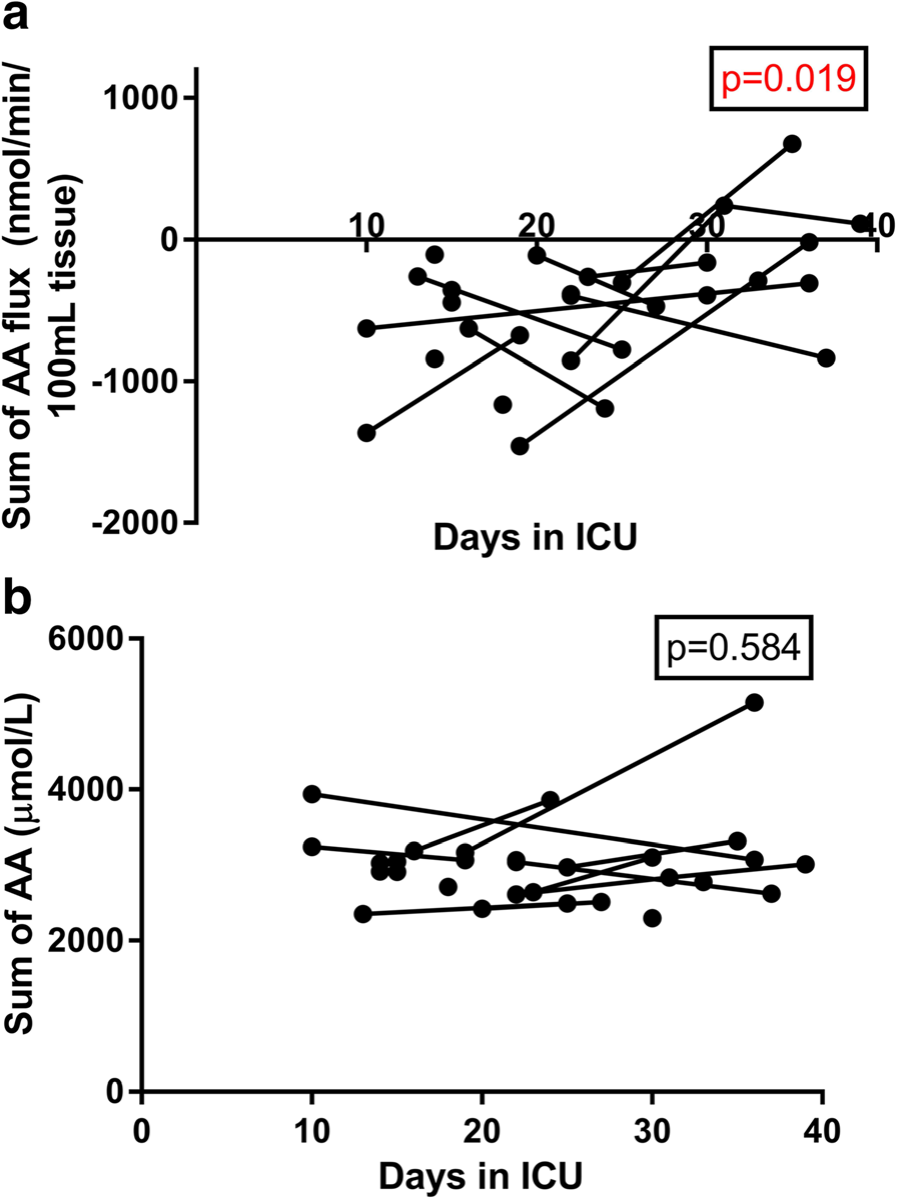
A longitudinal presentation of total free amino acids (AA) in plasma on 30 occasions in long-staying intensive care unit (ICU) patients (*n* = 20). **a** The fluxes across the leg. **b** The total free amino acid concentrations in arterial plasma. A negative flux indicates a net release from muscle tissue, and a positive flux indicates a net uptake into skeletal muscle. The *p* values from the linear mixed effects model against time in the ICU are indicated

The Sequential Organ Failure Assessment score and the nutritional intake (both protein and energy) did not change over time between days 10 and 40 (Additional file 1: Figure S3). The pattern of free amino acid exchange across the leg showed a net export of all individual amino acids except for glutamate, which showed a net uptake. There was a clear pattern of a lower amino acid export per volume of leg tissue over time, which attained statistical significance in the mixed effects model for > 50% of the individual amino acids. The same pattern was seen for both essential and nonessential amino acids. This is illustrated in Fig. 4a for the total sum of free amino acids. The fluxes of all the individual amino acids are displayed as Additional file 1: Figure S4. The total sum of arterial plasma concentrations of amino acids showed no change over time, as illustrated in Fig. 4b. In fact, none of the individual plasma free amino acid concentrations in the artery changed over time in the ICU (Additional file 1: Figure S5).

## Discussion

The novel observation in this study is an attenuation of leg muscle protein loss in terms of a less negative balance over time between days 30 and 40 of the ICU stay than in the initial phase of the ICU stay. This attenuation is most likely the result of an increasing muscle protein synthesis rate over time that is not significant in the longitudinal analyses but is in the two group analyses. This indicates that findings from the initial 10 days of the ICU stay cannot be extrapolated beyond this period. This observation is not without problems, however.

Studies of leg muscle turnover involving both de novo synthesis and breakdown rates in parallel require measurement of leg blood flow. In the present study, leg venous occlusion plethysmography was used. A crucial underlying assumption is that leg blood flow is not altered between measurements. This requirement was fulfilled on a group level. The results of muscle protein turnover as well as amino acid flux are commonly related to the volume of leg tissue. It is obvious that in a state of loss of both muscle tissue and muscle protein, the protein component of leg volume diminishes over time. In addition, leg or muscle edema can increase leg tissue volume but not the amount of muscle protein. It is difficult to adjust for this limitation. However, considering that the changes in muscle protein balance were to the largest degree attributable to increases in protein synthesis rates, whereas the rate of protein breakdown did not change over time, we argue that the changes observed were not due to changes in the leg volume composition and were due to real changes in the turnover rates.

The attenuation of the negative muscle protein balance of leg muscle observed over time is open to several possible interpretations. There may be an individual adaptation over time, possibly related to the nutritional intake and mobilization efforts. An alternative hypothesis is that the adaptation is present only on a group level and that those patients not able to maintain a negative muscle protein balance do not survive and are therefore not studied. However, owing to its observational nature, our study can neither confirm nor rule out this explanation.

All existing techniques to assess muscle protein losses in the critically ill are open to criticism. Originally, our working group published data on alkali-soluble protein content related to DNA [5]. Both these analyses have a large coefficient of variation related primarily to sample preparation, and the laboratory measurements are consequently highly investigator-dependent. Most data available in the literature are on skeletal muscle mixed protein synthesis, with results presented as fractional synthesis rates [7, 12, 14, 16]. The issue of the “precursor pool problem” has never been finally settled, although the study by Caso et al. clearly demonstrated that the equilibration into the precursor pool in muscle must be considered when protein synthesis rate is measured by incorporation of labeled amino acids into proteins [21]. In the present study, muscle protein synthesis rates were measured using the arteriovenous approach, and they were in the same range as previous findings showing rates that are normal or higher than normal [12]. So far, the only technique assessing breakdown rate is the technique used in the present study [10, 12]. A limitation is that both muscle synthesis and breakdown measurements rest on the same underlying assumptions, with the above-mentioned possible alteration of tissue composition in the leg as an example.

Assessments of muscle protein synthesis and breakdown rates have the potential to pick up small changes that will affect the protein balance and eventually muscle protein mass. However, these are complicated and invasive to perform, making them less suitable for guiding daily clinical treatment. Therefore, measurements of muscle mass by imaging are welcomed as a valuable complement. There are two different approaches used in the ICU setting: ultrasound and CT. Both techniques are subject to very rapid technical development. The principal difficulties of both techniques are their resolution and the variable water content of muscle tissue. It is also obvious that both the bedside handling and the interpretation are extremely open to criticism for being investigator-dependent. A recent study using ultrasound shows that the loss of muscle thickness in ICU patients tends to level out around day 30 [22]. This agrees with our data showing that the net protein balance is close to zero from day 30 onward.

The kinetics of the individual amino acids across the leg showed a rather uniform pattern. First, there was a constant export of amino acids, essential as well as nonessential, except for glutamate, which was constantly taken up by the leg. This loss of amino acids was in spite of ongoing nutritional support during the measurement periods. The second pattern was the clear tendency of a less pronounced amino acid export over time, which was remarkably uniform among the individual amino acids. The documentation of no change in plasma flow strengthens this observation. The attenuation of skeletal muscle net protein balance and the attenuation of the free amino acid efflux from the leg obviously are in parallel with the protein turnover results as supportive observations.

## Conclusions

We present data on skeletal muscle mixed protein turnover in patients with a prolonged ICU stay. This is a unique observation, and although the interpretation of data is not without problems, it is fair to conclude that observations in the initial 10 days of critical care cannot automatically be extrapolated to a prolonged ICU stay. On the contrary, the present data show an adaptation over time, at least among survivors (both short-term and long-term). The heterogeneity of the cohort of patients assembled does not, in our opinion, invalidate this conclusion.

## Additional file

Additional file 1: **Table S1.** Individual patients characteristics. **Table S2.** Patient characteristics at the two measurement points. **Figure S1.** Longitudinal presentation of plasma flow. **Figure S2.** Longitudinal presentation of phenylalanine turnover using the two-pool model. **Figure S3.** Longitudinal presentation of SOFA scores and nutrition. **Figure S4.** Longitudinal presentation of all amino acid fluxes. **Figure S5.** Amino acid concentrations. (PDF 185 kb)

## Abbreviations

AA: Amino acids
BMI: Body mass index
CT: Computed tomography
ICU: Intensive care unit
SOFA: Sequential Organ Failure Assessment
