# Supplementary material for: An attenuated rate of leg muscle protein depletion and leg free amino acid efflux over time is seen in ICU long-stayers
Source: Crit Care. 2018 Jan 23;22:13. doi: 10.1186/s13054-017-1932-6 (PMC5782367; doi:10.1186/s13054-017-1932-6)
Supplement: Additional file 1: — Table S1. Individual patients characteristics. Table S2. Patient characteristics at the two measurement points. Figure S1. Longitudinal presentation of plasma flow. Figure S2. Longitudinal presentation of phenylalanine turnover using the two-pool model. Figure S3. Longitudinal presentation of SOFA scores and nutrition. Figure S4. Longitudinal presentation of all amino acid fluxes. Figure S5. Amino acid concentrations. (PDF 185 kb) [file 13054_2017_1932_MOESM1_ESM.pdf]

Additional file 1 to:

An attenuated rate of leg muscle protein depletion and leg free amino acid efflux over time is seen in ICU long-stayers.

Lena Gamrin-Gripenberg[1], Martin Sundström Rehal[1], Daniel Olsson[2], Jonathan Grip[1], Jan Wernerman[1], Olav Rooyackers[1]

[1]Department of Anestesiology and Intensive Care Medicine, Perioperative Medicine and Intensive Care, Karolinska University Hospital Huddinge, and CLINTEC, Karolinska Institutet, Huddinge, Sweden.

[2]Medical Statistics, Department of Learning, Informatics, Management and Ethics, Karolinska Institutet, Stockholm, Sweden

Table S1. Individual patient characteristics.

Pat. no.	BMI[1]	ICU LOS	SOFA	Kcal/24h	Kcal/kg/24h	AA (g)/24h	AA (g)/kg/24h	Insulin
	(kg/m²)	(days)	median (IQR)	mean±SD	mean±SD	mean±SD	mean±SD	median (IQR)
1	27.9	42	6 (6-7)	1764±451	19±5	92±37	1.0±0.4	44 (22-80)
2	33.5	31	4 (3-6)	2096±460	19±4	98±25	0.9±0.2	179 (157-212)
3	27.0	30	8 (7-8)	1365±379	17±5	74±21	0.9±0.3	73 (52-99)
4	23.5	24	6 (5-8)	1479±306	20±4	83±17	1.1±0.2	51 (37-96)
5	25.4	36	4 (4-7)	1737±369	19±4	86±20	0.9±0.2	74 (57-101)
6	23.2	22	4 (3-6)	1513±403	20±5	76±25	1.0±0.3	70 (58-75)
7	30.5	17	4 (1-5)	1776±631	17±6	81±29	0.8±0.3	66 (26-73)
8	38.6	69	12 (10-13)	2138±401	14±3	105±23	0.7±0.1	87 (70-115)
9	24.7	30	5 (5-6)	1452±536	20±7	73±27	1.0±0.4	57 (32-78)
10	25.9	16	2 (2-3)	1314±491	17±6	74±29	0.9±0.4	31 (28-40)
11	28.4	27	8 (6-9)	1410±598	15±6	73±35	0.8±0.4	55 (24-85)
12	23.4	9	12 (11-13)	1149±526	19±9	64±32	1.0±0.5	27 (17-54)
13	23.7	21	7 (6-9)	1540±338	23±5	77±19	1.2±0.3	70 (34-90)
14	19.4	24	6 (4-9)	1152±374	22±7	65±32	1.2±0.6	5 (3-6)
15	22.0	27	4 (2-4)	1854±561	24±7	98±32	1.3±0.4	81 (72-101)
16	33.8	24	7 (3-7)	1925±678	18±6	99±35	0.9±0.3	39 (26-49)
17	15.7	14	6.5 (5.5-7)	1272±650	22±11	50±35	0.8±0.6	28 (22-47)
18	32.2	21	3 (3-3)	1764±493	17±5	81±25	0.8±0.2	43 (30-45)
19	26.1	68	3 (2-3)	2410±800	23±8	114±39	1.1±0.4	22 (18-26)
20	27.4	88	6 (4-9)	2269±519	27±6	99±24	1.2±0.3	24 (16-30)

[1]: mean of study days. AA: Amino acids; ICU: Intensive care unit; IQR: Inter-quartile range; LOS: Length of stay; SOFA: Sequential Organ Failure Assessment. Data is presented as mean ± SD for normally distributed data and as median (IQR) for non-normally distributed data.

Table S2. Patients characteristics at the 2 measurement periods illustrated in Figure 2.

	ICU day 10-20	ICU day 30-40
**Weight (kg)**	75 (64, 86)	78 (70, 91)
**SOFA**	7 (5, 8)	5 (4, 7)
**Mechanical ventilation (%)**	100 %	100%
**CRRT (%)**	30 %	30 %
**Caloric intake[1] (kcal/kg/day)**	21 (19, 25)	18 (18, 23)
**Amino acid intake[1] (g/kg/day)**	1.1 (1.0, 1.2)	1.0 (0.9-1.2)
**Insulin IU/24 h**	45 (27, 86)	26 (13, 69)

Values are given as n(%), medians (inter-quartile range). [1]: Median during 5 days before sampling.

Figure S1.

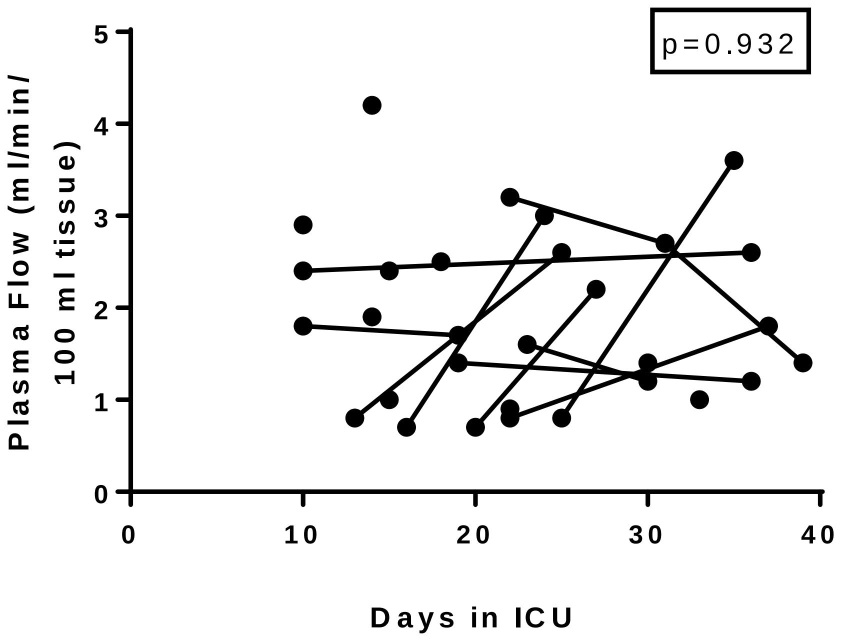

Figure S1. A longitudinal presentation of plasma flow across the leg on 30 occasions measured by venous occlusion plethysmography in long-staying ICU patients (n=20). The p-values from the linear mixed effect model against time in the ICU are indicated.

Figure S2.

A.

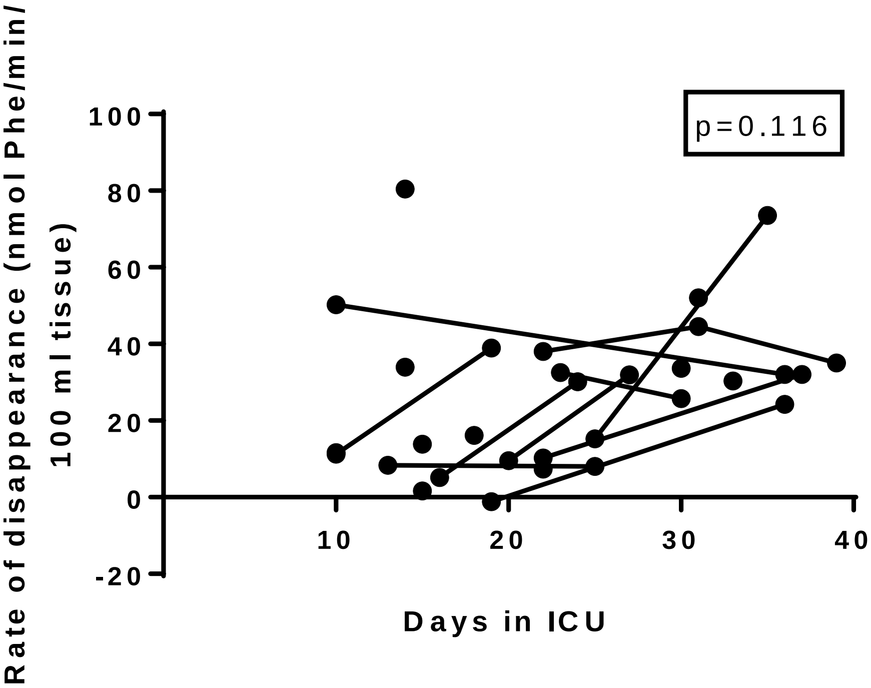

B.

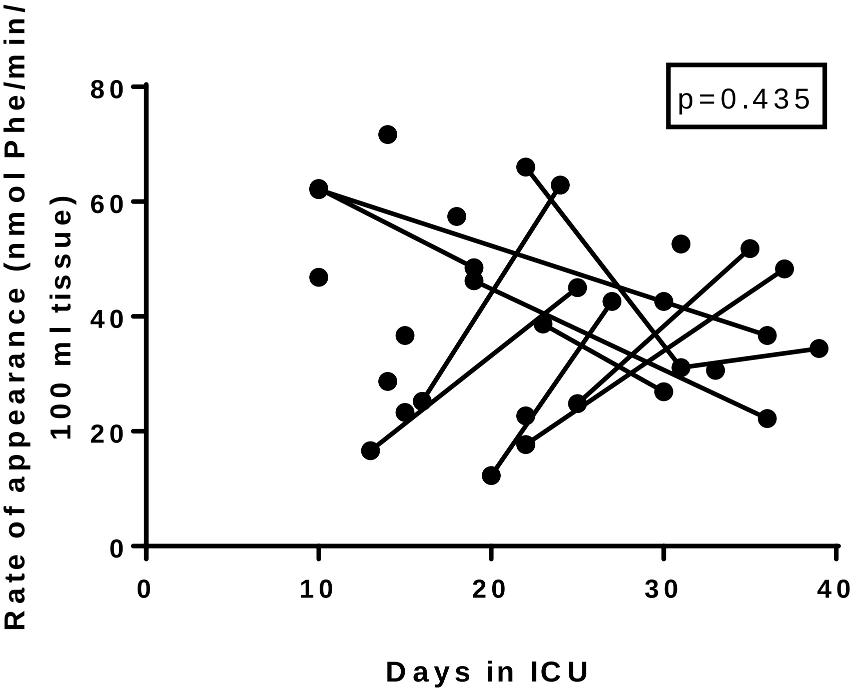

Figure S2. A longitudinal presentation of phenylalanine turnover in the leg on 30 occasions in long-staying ICU patients (n=20) assessed with a 2-pool model. The two panels depict; A) the rate of disappearance, and B) the rate of appearance. The p-values from the linear mixed effect model against time in the ICU are indicated.

Figure S3.

A.

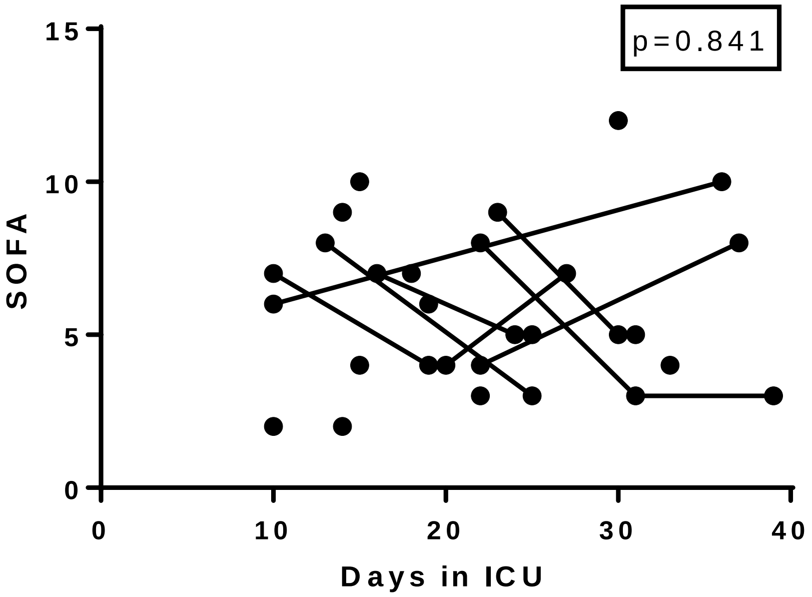
p=0.841
SOFA
15
10
5
0
0
10
20
30
40
Days in ICU

B.

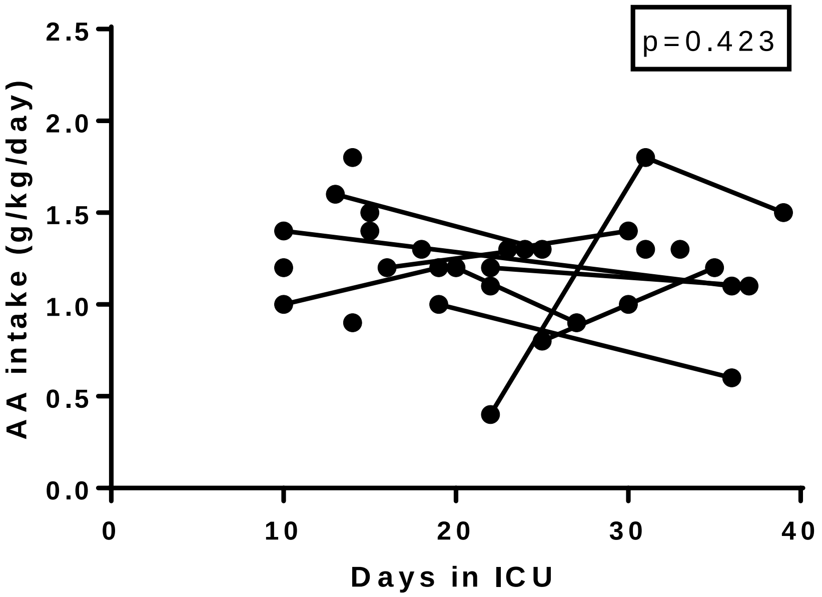
p=0.423
AA intake (g/kg/day)
2.5
2.0
1.5
1.0
0.5
0.0
0
10
20
30
40
Days in ICU

C.

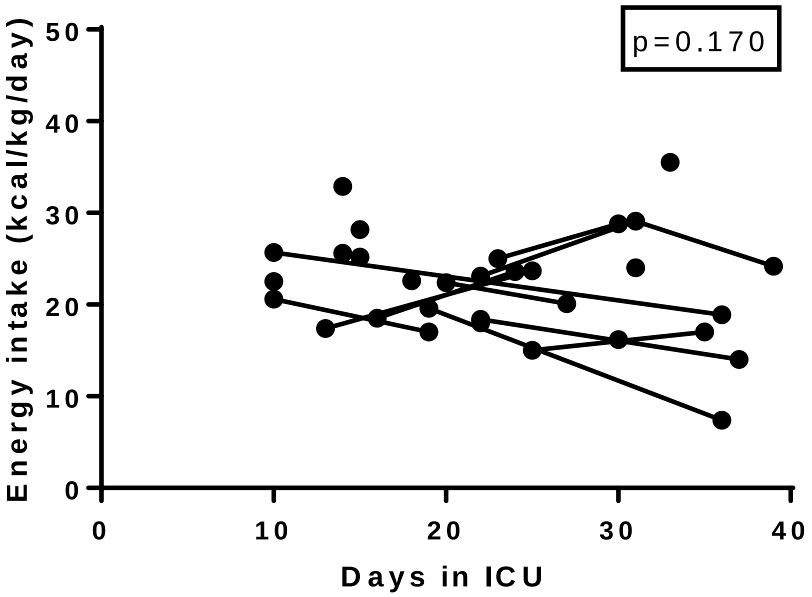

Figure S3. A longitudinal presentation of the SOFA score (panel A), the amino acid intake (panel B) and the energy intake (panel C) on 30 occasions measured in long-staying ICU patients (n=20). The p-values from the linear mixed effect model against time in the ICU are indicated.

Figure S4. Longitudinal presentations of free amino acid fluxes across the leg on 30 occasions in longstaying ICU patients (n=20). Individual amino acids are displayed in 21 panels, in addition also the combined branched chain amino acids (BCAA) and the combined essential amino acids (EAA) are displayed in two additional piles. The p-values from the linear mixed effect model against time in the ICU are indicated. Significant correlations are indicated in red text.

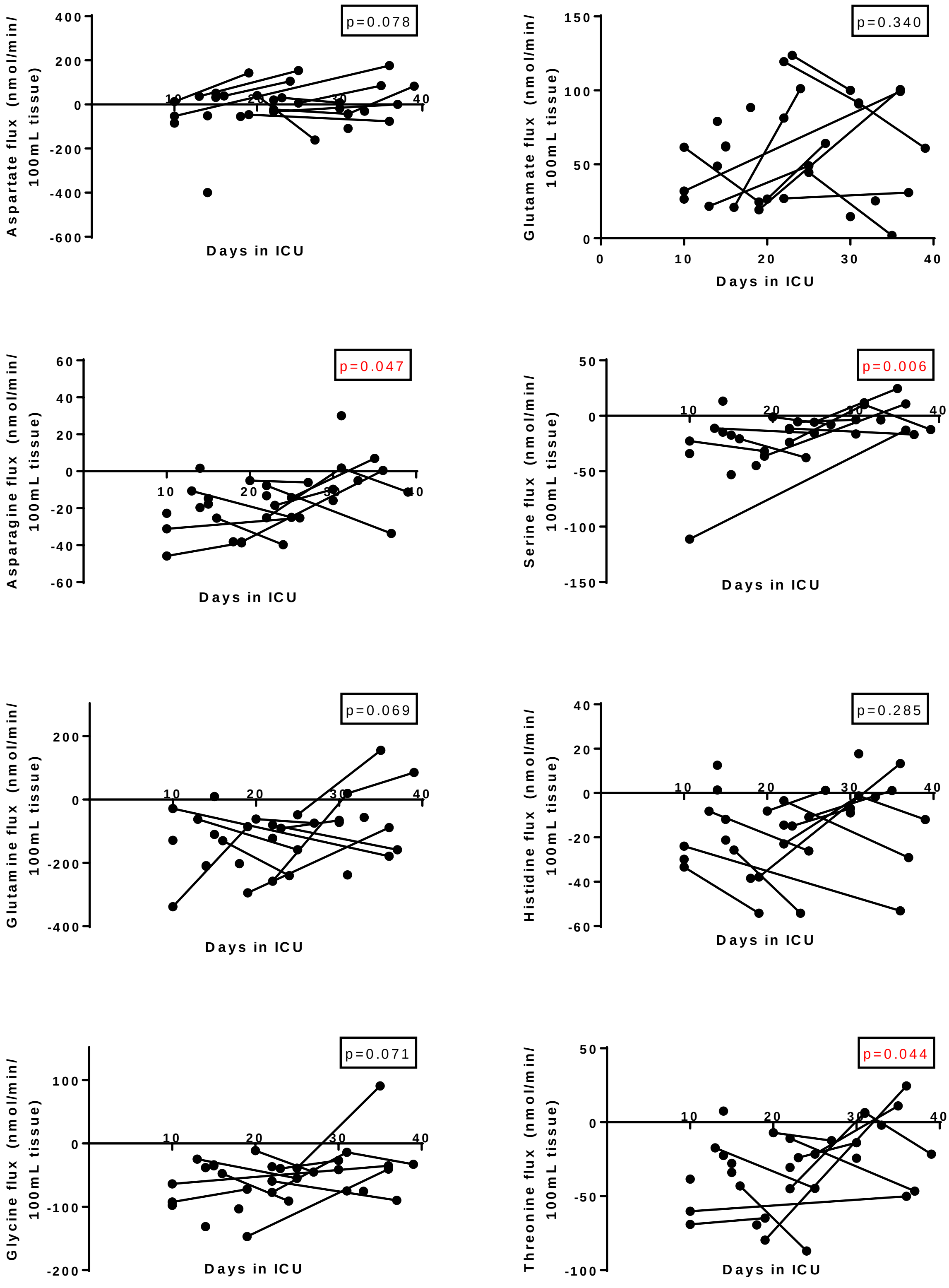

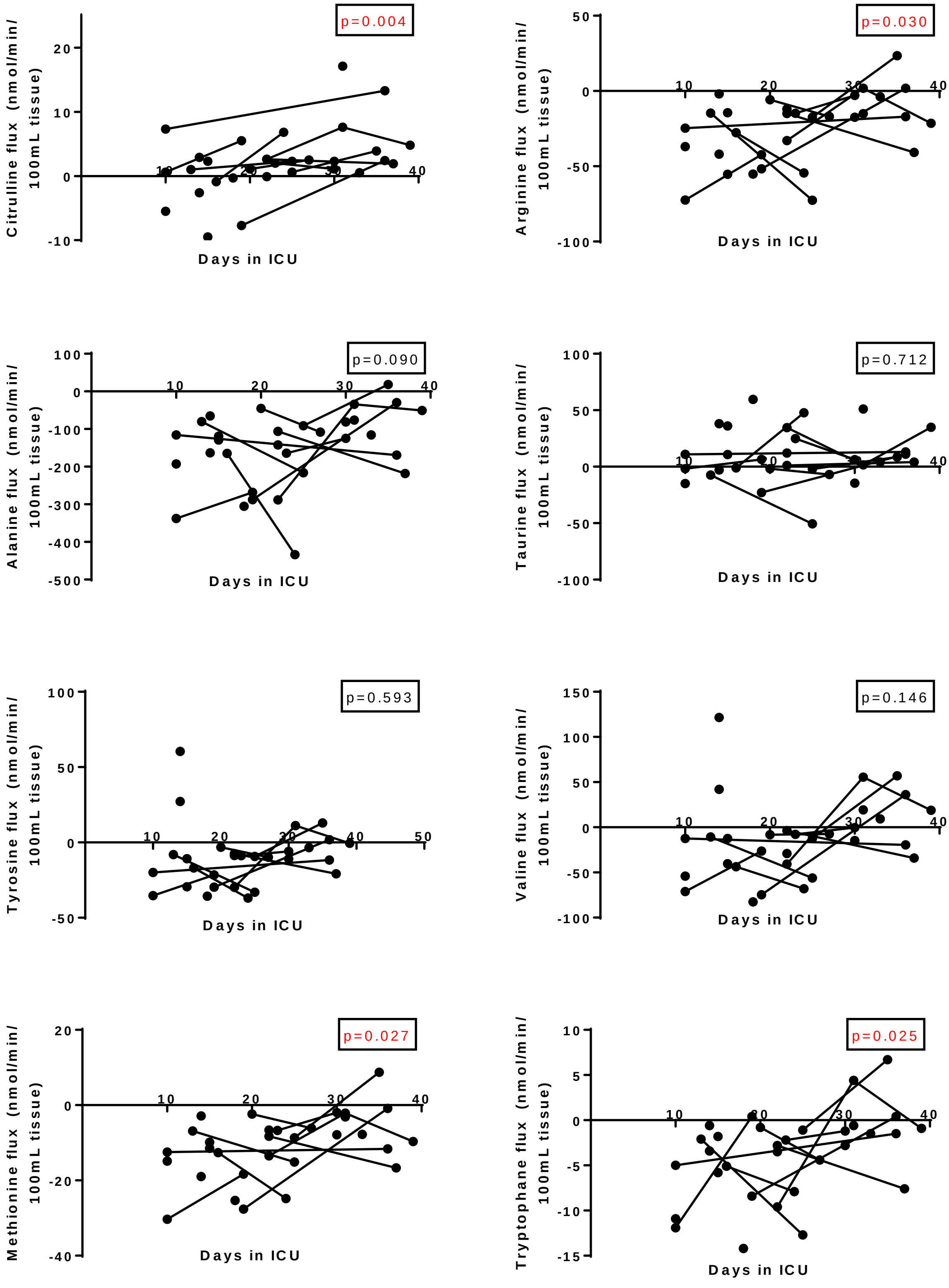

p=0.004
Citrulline flux (nmol/min/ 100mL tissue)
Days in ICU
p=0.030
Arginine flux (nmol/min/ 100mL tissue)
Days in ICU
p=0.090
Alanine flux (nmol/min/ 100mL tissue)
Days in ICU
p=0.712
Taurine flux (nmol/min/ 100mL tissue)
Days in ICU
p=0.593
Tyrosine flux (nmol/min/ 100mL tissue)
Days in ICU
p=0.146
Valine flux (nmol/min/ 100mL tissue)
Days in ICU
p=0.027
Methionine flux (nmol/min/ 100mL tissue)
Days in ICU
p=0.025
Tryptophane flux (nmol/min/ 100mL tissue)
Days in ICU

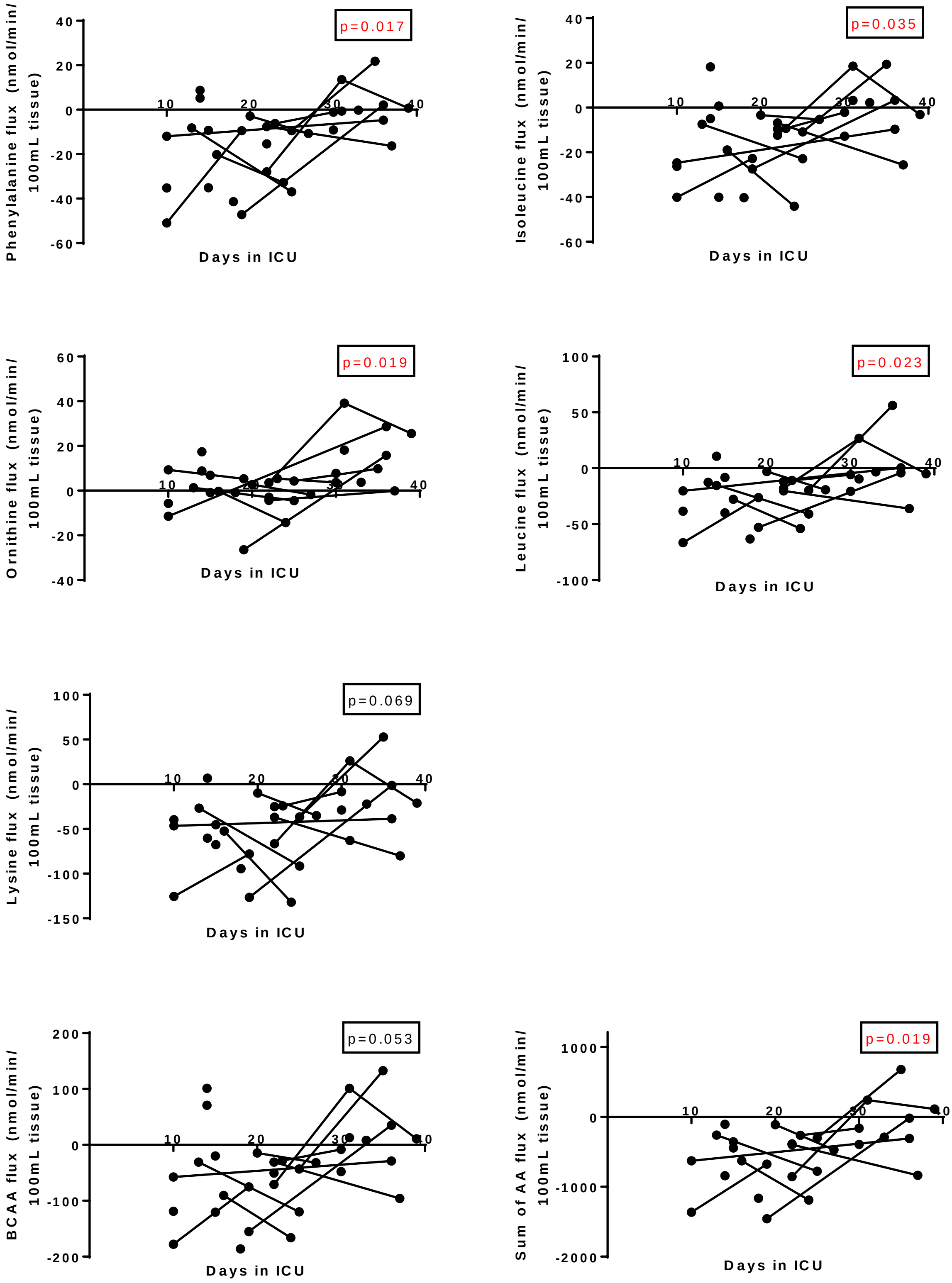
Phenylalanine flux (nmol/min/ 100mL tissue)
p=0.017
Days in ICU
Isoleucine flux (nmol/min/ 100mL tissue)
p=0.035
Days in ICU
Ornithine flux (nmol/min/ 100mL tissue)
p=0.019
Days in ICU
Leucine flux (nmol/min/ 100mL tissue)
p=0.023
Days in ICU
Lysine flux (nmol/min/ 100mL tissue)
p=0.069
Days in ICU
BCAA flux (nmol/min/ 100mL tissue)
p=0.053
Days in ICU
Sum of AA flux (nmol/min/ 100mL tissue)
p=0.019
Days in ICU

Figure S5. Longitudinal presentations of free amino acid concentrations in arterial plasma on 30 occasions in longstaying ICU patients (n=20). Individual amino acids are displayed in 21 panels, in addition also the combined branched chain amino acids (BCAA) and the combined essential amino acids (EAA) are displayed in two additional piles. The p-values from the linear mixed effect model against time in the ICU are indicated. Significant correlations are indicated in red text.

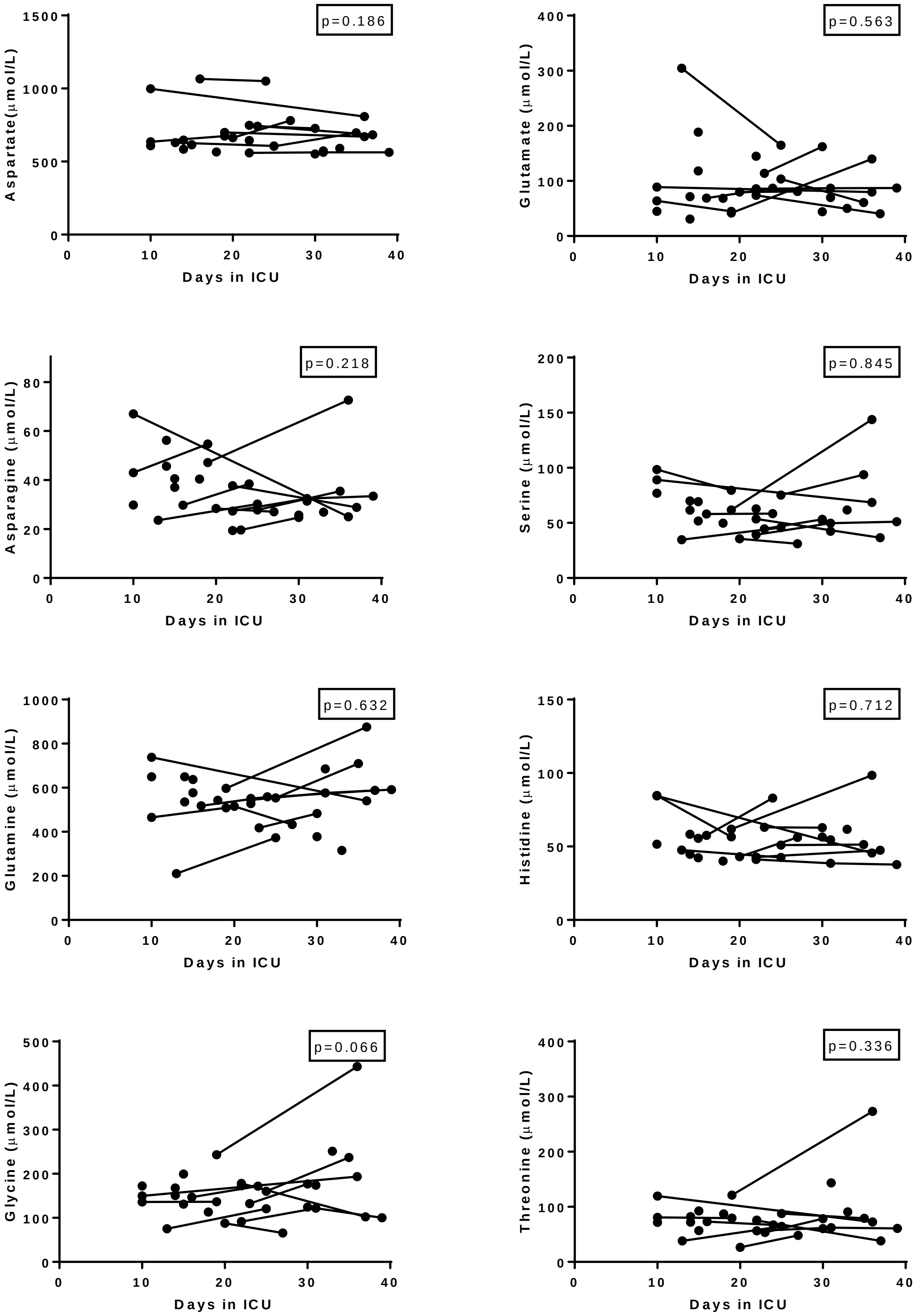

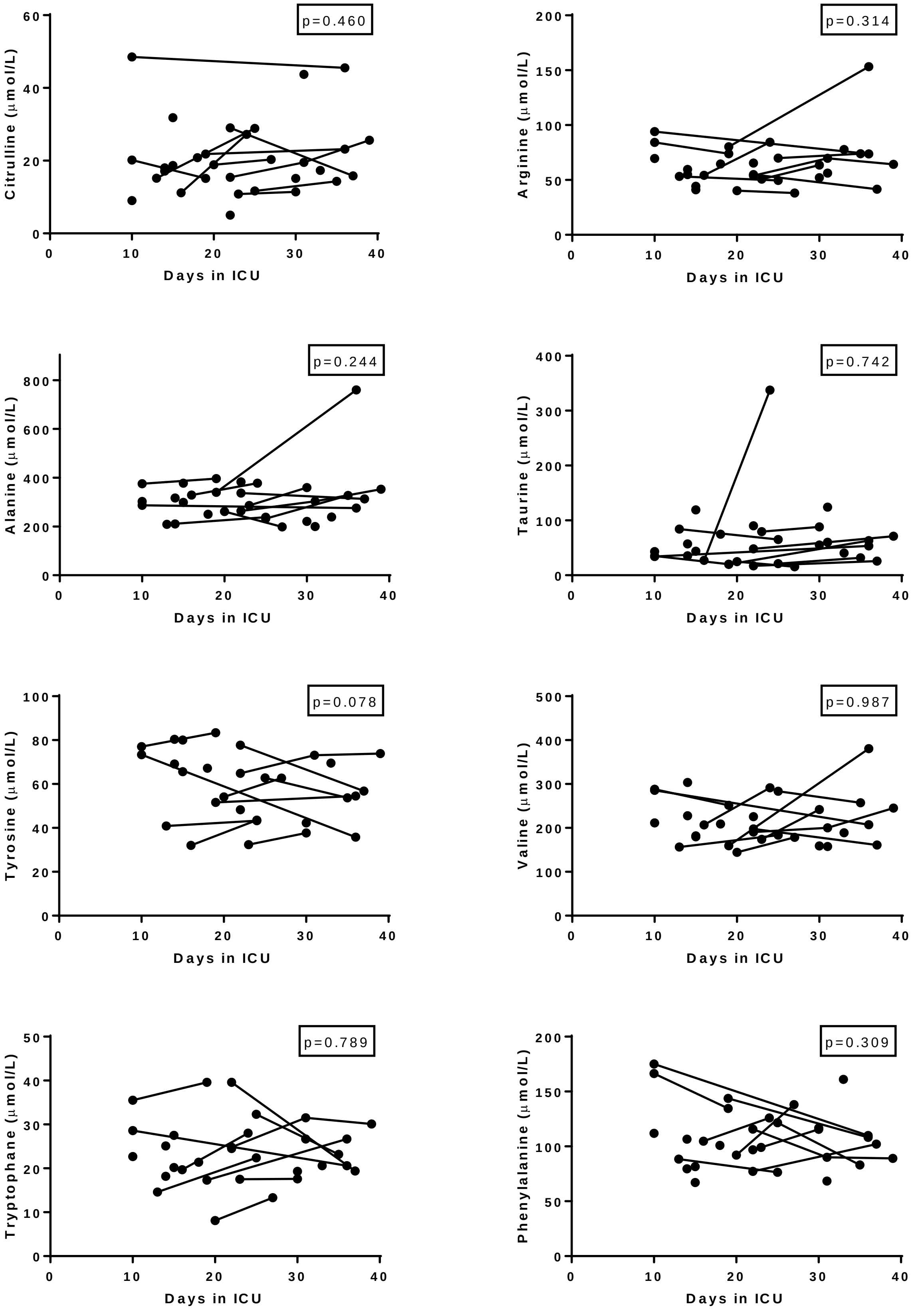
p=0.460
Citrulline (µmol/L)
Days in ICU
p=0.314
Arginine (µmol/L)
Days in ICU
p=0.244
Alanine (µmol/L)
Days in ICU
p=0.742
Taurine (µmol/L)
Days in ICU
p=0.078
Tyrosine (µmol/L)
Days in ICU
p=0.987
Valine (µmol/L)
Days in ICU
p=0.789
Tryptophane (µmol/L)
Days in ICU
p=0.309
Phenylalanine (µmol/L)
Days in ICU

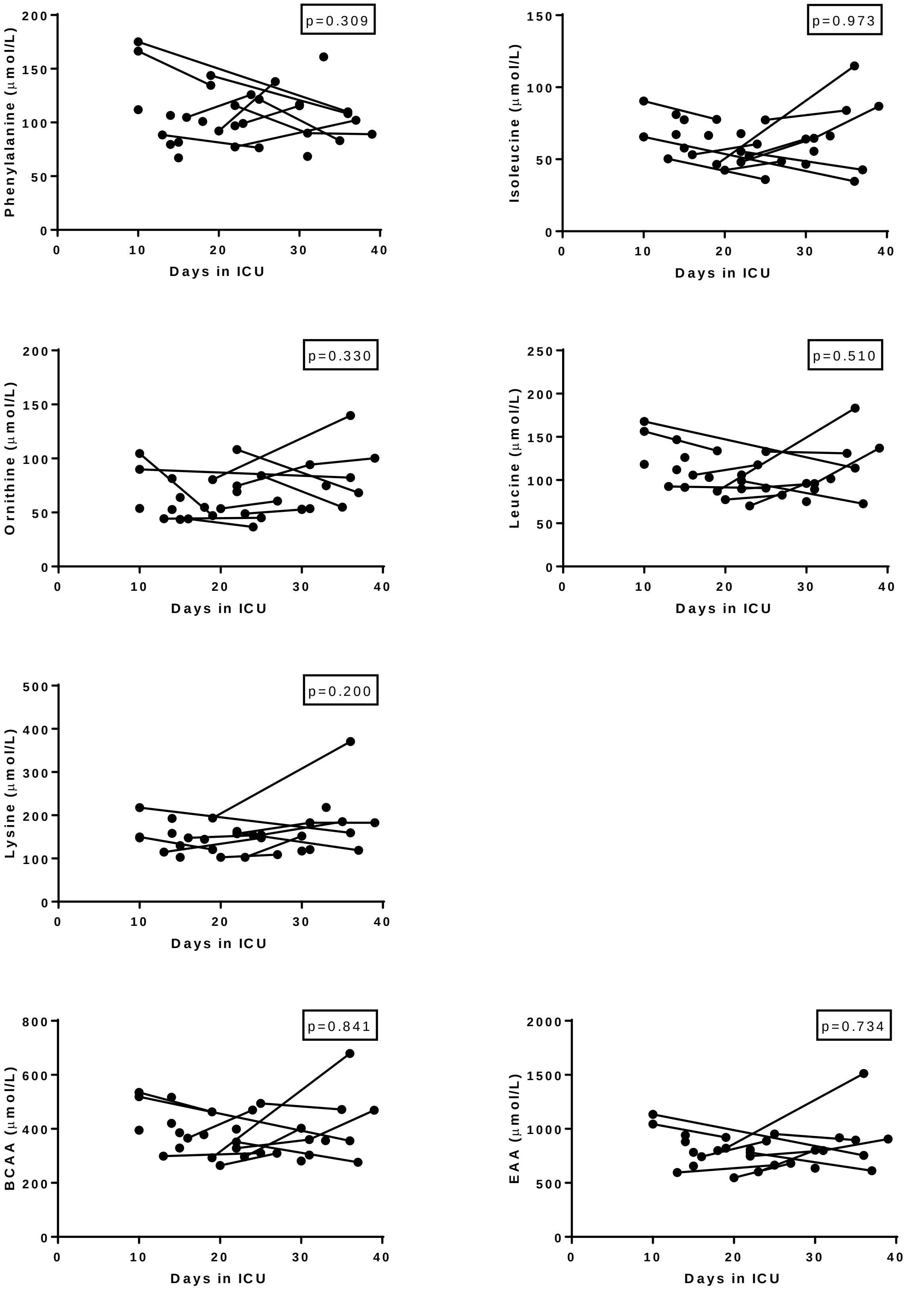
Phenylalanine (µmol/L)
p=0.309
Isoleucine (µmol/L)
p=0.973
Ornithine (µmol/L)
p=0.330
Leucine (µmol/L)
p=0.510
Lysine (µmol/L)
p=0.200
BCAA (µmol/L)
p=0.841
EAA (µmol/L)
p=0.734
Days in ICU
